# Supplementary material for: Whole-exome sequencing analysis identifies risk genes for schizophrenia
Source: Nat Commun. 2025 Aug 2;16:7102. doi: 10.1038/s41467-025-62429-y (PMC12318047; doi:10.1038/s41467-025-62429-y)
Supplement: Supplementary file 1 — Supplementary Information [file 41467_2025_62429_MOESM1_ESM.pdf]

Supplementary Information: Whole-exome sequencing analysis identifies risk genes for schizophrenia

Table of Contents

Supplementary Methods 2

Sample description 2

Sample-level quality control 3

Genotype-level quality control 8

Variant-level quality control 8

Variant annotation 9

Analysis of SCHEMA rare coding variant data 9

Gene set analysis in the new case-control sample 11

Analysis of previously implicated genes in the new case-control sample 11

Supplementary Note 1 12

Relationship between sequencing depth and burden of singleton coding variants 12

Supplementary Note 2 15

Summary of novel schizophrenia FDR < 5% genes 15

Supplementary Note 3 18

Expression profiles of genes implicated in schizophrenia at exome-wide significance and FDR < 5% 18

Expression in adult human tissue 18

Developmental expression in 6 human brain regions 18

Cellular expression in fetal and adult human frontal cortex 21

Supplementary Tables 5-11 23

Supplementary Table 5: 23

Supplementary Table 6 24

Supplementary Table 7 25

Supplementary Table 8. 26

Supplementary Table 9. 27

Supplementary Table 10. 28

Supplementary Table 11. 29

Supplementary Figure 10 30

Supplementary References 31

Supplementary Methods

Sample description

Schizophrenia cases

4,482 of the new cases were from the CLOZUK2 cohort[1]. Anonymised blood samples from CLOZUK2 were ascertained from patients receiving clozapine and having a clinical diagnosis of treatment-resistant schizophrenia. A validation for using a clinician diagnosis of treatment-resistant schizophrenia against a research diagnostic criteria for schizophrenia can be found in Supplementary Note (2) of Pardiñas et al 2018[1]. The new sample also included the following clinically ascertained cohorts: CardiffCOGS (n = 429)[2,3], F-series (n= 453[4]), and Affected-Sib (n = 161; NB only 1 affected member of each sib-pair was included[5]). All clinically ascertained cases meet DSMIV[6] or ICD10[7] criteria for schizophrenia or schizoaffective disorder. Further details on these clinically ascertained samples can be found in the respective published studies cited above.

Controls

The new controls were derived from the following cohorts: **1)** 1,595 WTCCC2 controls from the 1958 birth cohort[8,9]; **2)** 398 NCMH controls that were recruited by the National Centre for Mental Health at Cardiff University and screened for the presence of psychiatric disorders[10]; **3)** 5,275 Cardiff Alzheimer's disease (AD) cohort samples from a recent exome-sequencing project on dementia conducted by Cardiff University. 80% of this sample comprises individuals with AD. A subset of these are included in Holstege et al. 2022, which contains further cohort descriptives. We included individuals with AD in our new control sample given the evidence that in genetic studies of relatively uncommon disorders, the increased power afforded by larger samples of controls, even completely unscreened controls, is expected to be more than offset by the accidental inclusion of cases in that larger control sample[11]. We also note that while some studies have reported a very weak positive genetic association between schizophrenia and AD (r2 0.03-0.1)[12], the evidence is inconsistent[13,14]. Moreover, to the best of our knowledge,

no studies have been published demonstrating a rare variant overlap between AD and schizophrenia.

Sample-level quality control

Initial sample exclusions and sex checks: 28 samples were excluded for having a sample call rate < 0.75 or a mean genotype depth < 10. An additional 37 cases and 4 controls were excluded for failing sex checks, where sample sex as imputed using Peddy[15] did not match their recorded sex. We were unable to perform sex checks on WTCCC2 controls as we did not have access to their recorded sex.

Relatedness exclusions: Hail's PC-Relate method (https://hail.is/docs/0.2/index.html) was used to estimate pairwise kinship coefficients (Φ_{ij}) between all pairs of samples, based on linkage disequilibrium (LD) pruned SNPs (max $r^2 < 0.1$) with a MAF > 0.01 and a variant call rate > 0.98. The first 2 principal components (PCs) were included to correct for population structure in the kinship calculation. Pairs of individuals where $\Phi_{ij} \geq 0.45$ were considered duplicates or monozygotic twins, those with Φ_{ij} between 0.1 and 0.4 and identity-by-descent 0 (ibd0) < 0.1 as parent-child relatives, those with Φ_{ij} between 0.1 and 0.4 and ibd0 between 0.1 and 0.4 as siblings, those with Φ_{ij} between 0.1 and 0.2 and ibd0 between 0.4 and 0.7 as 2nd degree relatives, and those with Φ_{ij} between 0.1 and 0.2 and ibd0 > 0.7 as 3rd degree relatives (Supplementary Figure 1). We excluded related individuals to ensure that no two samples were third-degree or closer in relationship, prioritising the retention of schizophrenia samples. This resulted in the exclusion of 224 cases and 331 controls.

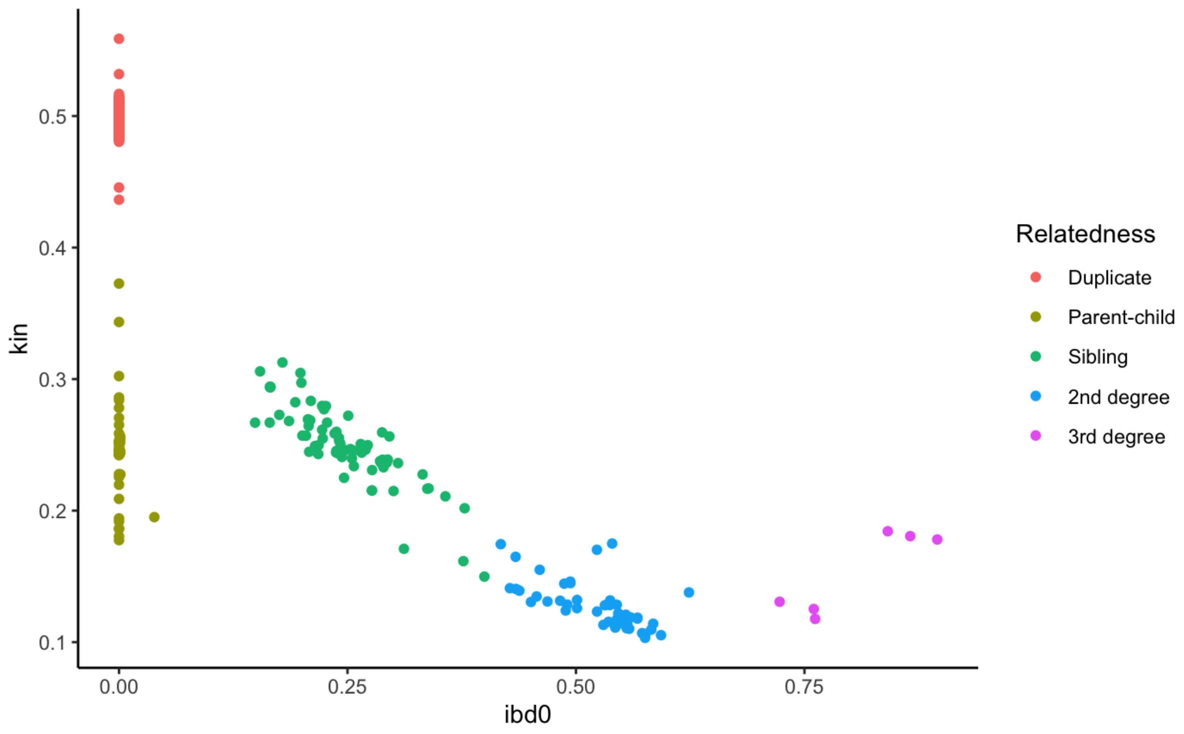

Supplementary Figure 1. Sample relatedness in the new case-control dataset. Estimated kinship coefficients (kin) and proportion of identity-by-descent zero (ibd0) alleles for each pair of individuals with a kinship coefficient greater than 0.1. Kin and IBD0 statistics were generated using Hail's PC-Relate method[16].

Controlling for population structure: Individuals in the new case-control sample were first grouped based on genetic similarity to the superpopulations used in the 1000 genomes (1KG) project. Here, Peddy[15] was used to perform a principal components (PC) analysis and then train a support vector machine (SVM) on the first 4 PCs derived from samples in the 1KG project, using the 1KG sample superpopulations as training labels (Supplementary Figure 2 A). To control for population structure, we first excluded 140 cases and 36 controls that fell 4 standard deviations or more from the means of PCs 1 and 2 in samples predicted by Peddy to be genetically similar to the 1KG European superpopulation reference (Supplementary Figure 2 A). We then applied Hail's hwe_normalized_pca function to the remaining samples, which identified two PC-associated clusters (Supplementary Figure 2 B). As shown in Supplementary Figure 2 B, the majority of samples were observed in cluster 1, with cluster 2 containing only 5.5% of cases and 18.1% of controls. The mean number of singleton variants carried by

samples within the boundaries of PCA-cluster 1 as shown in Supplementary Figure 2 B (mean n = 88.3; standard error (SE) = 0.23) was less than half of that observed for samples outside of this cluster (mean n = 210.2; SE = 1.55). To ensure our findings are not confounded by population structure, we restricted our analysis to samples within the boundaries of PCA-cluster 1 as shown in Supplementary Figure 2 B. This resulted in the exclusion of an additional 278 cases and 892 controls.

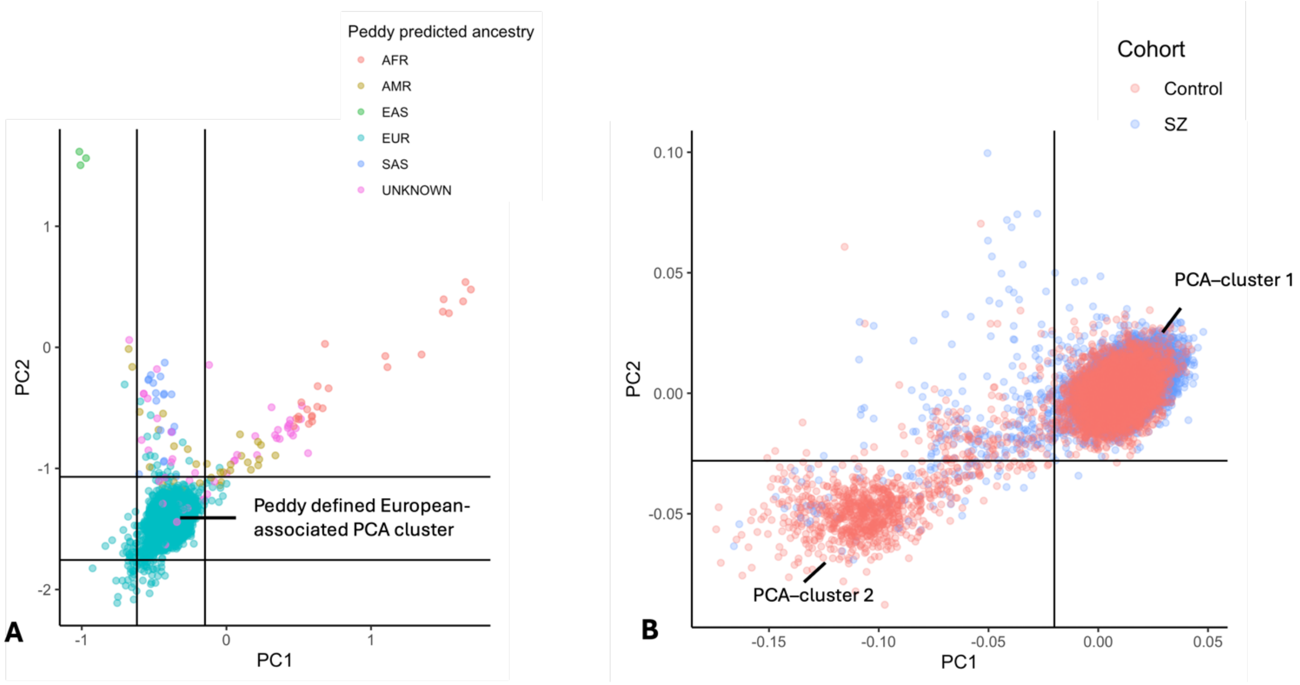

Supplementary Figure 2. A) Principal component analysis and sample ancestry as predicted by Peddy[15]. Horizontal and vertical lines indicate 4 standard deviations from the mean of PC1 and PC2 in samples defined by Peddy as belonging to the European-associated PCA cluster. ARF = African; AMR = Ad Mixed American; EAS = East Asian; EUR = European; SAS = South Asian **B)** Principal component analysis for samples in the Peddy-defined European-associated PCA cluster (see Figure 3A). To control for population structure, samples outside of the boundaries of PCA-cluster 1 were excluded.

Hard filters: For samples passing all quality control described above, we applied Hail's sample_qc function to variants passing genotype and variant QC (described below), and excluded samples based on the following filters: call rate < 0.96; number of singletons > 110; TiTV ratio < 2.95 or > 3.15; het/hom ratio < 1.43 or > 1.82

(Supplementary Figure 3). A total of 232 samples were excluded after applying hard filters.

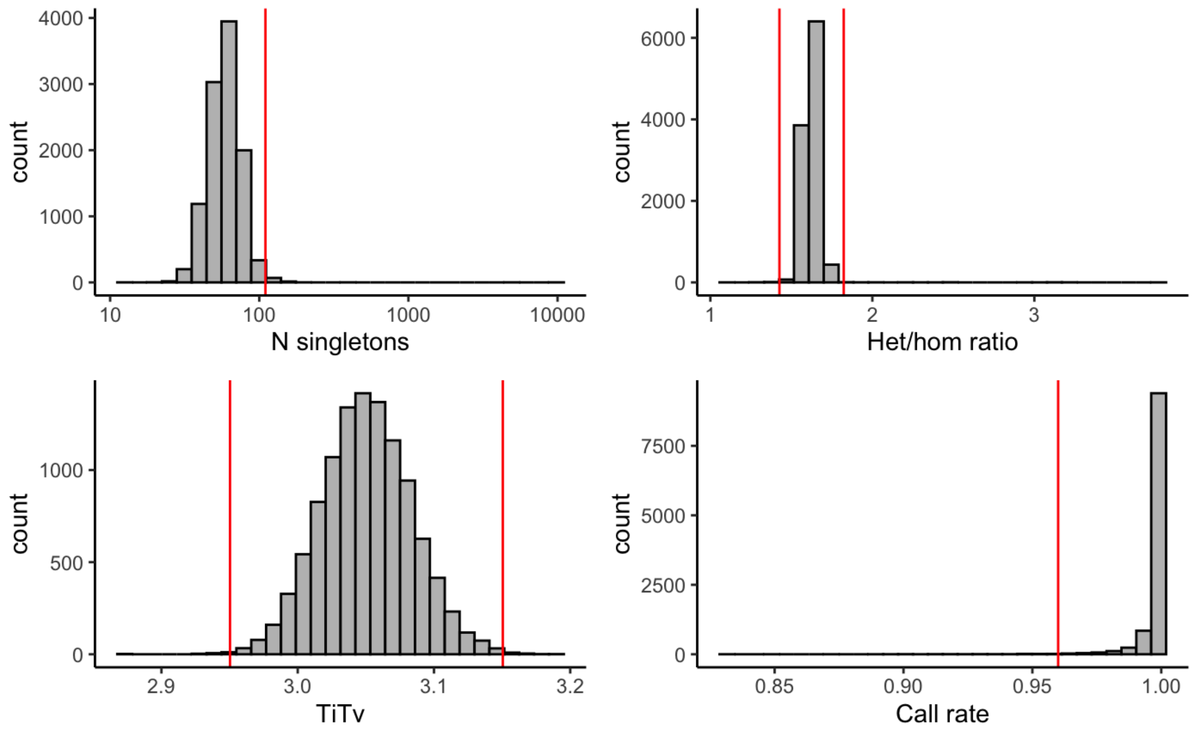

Supplementary Figure 3. Distributions of sample-level QC metrics in new case-control sample. Histograms show distribution of the number of singleton variants, heterozygous/homozygous variant ratio, transition/transversion ratio, and call rate per sample. Red lines indicate the threshold used to filter samples that failed 'hard filter' QC.

Exclusion of samples overlapping with SCHEMA study: To ensure that the new sample is independent from cases we previously contributed to the SCHEMA study[17], we performed an IBD analysis using microarray data from all schizophrenia datasets held by Cardiff University (CLOZUK 1, 2 and 3, CardiffCOGs, F-series and affected-sibpair samples), and excluded 6 cases in our new exome-sequencing sample found to be a duplicate (PI-HAT value > 0.9) with a case included in the SCHEMA study. Additionally, for each new case/control sample with a rare variant in the 12 previously implicated exome-wide significant schizophrenia genes, or in any of the novel risk genes identified in the current study, we examined the percentage of singleton coding variants

carried across the exome that are also observed in SCHEMA cases, which we determined using the published variant-level data from the SCHEMA study. The mean percentage of singleton variants carried by these samples that are also observed in SCHEMA was 22.7%, with two cases being clear outliers with > 75% of their singleton variants also observed in SCHEMA (Supplementary Figure 4). To ensure that our primary findings are not influenced by sample overlaps, we excluded these two cases from our study.

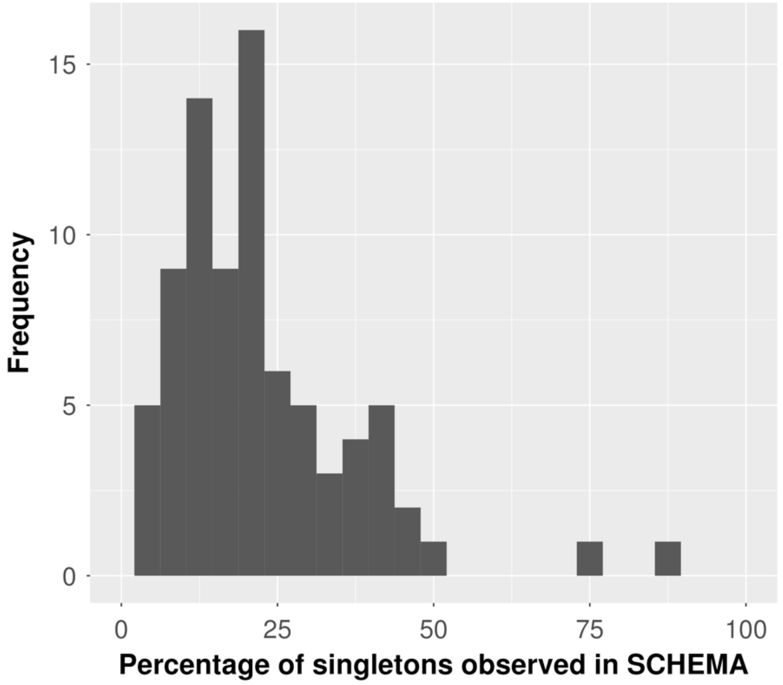

Supplementary Figure 4. Percentage of variants per-sample observed in SCHEMA. For each of the new samples with a rare variant in the 12 previously implicated exome-wide significant schizophrenia genes, or in any of the novel risk genes identified in the current study, the percentage of singleton coding variants carried across the exome that are also observed in SCHEMA cases is shown.

Contamination: For samples passing the above QC, we estimated sequencing contamination using the compute_charr method in Hail[18]. All samples had < 5% estimated contamination. Thus, no samples were excluded for having excess contamination.

Summary of all sample quality control

After applying all sample-level quality control described above, 4,650 cases and 5,719 controls were retained for analysis. A hierarchical summary of sample exclusions is provided in Supplementary Table 1.

	**Control**	**Schizophrenia**
Count pre-QC	7,268	5,525
Initial sample exclusions	4	24
Sex check exclusions	133	37
Relatedness exclusions	331	224
Ancestry exclusions	1,001	418
Hard filter exclusions	80	164
SCHEMA sample overlaps	0	8
**Passed samples**	**5,719**	**4,650**
**Passed males**	**2,729**	**3,329**
**Passed females**	**2,990**	**1,321**

Supplementary Table 1. Hierarchical summary of quality control sample exclusions in the new case-control sample.

Genotype-level quality control

Genotype calls were defined as low quality and excluded if they met any of the following criteria: total depth < 10x; genotype quality score < 20; allele balance > 0.1 and < 0.9 for homozygous genotypes for the reference and alternative allele, respectively; allele balance < 0.25 or > 0.75 for heterozygous genotypes. For variants falling within the X chromosome non-pseudoautosomal (non-PAR) region, male heterozygote genotypes were excluded.

Variant-level quality control

Variant sites were defined as low quality and excluded if they met any of the following criteria: a multi-allelic site with > 6 alternative alleles; in a region of low sequence

complexity[19]; failed GATK VQSR filters; not in Hardy-Weinberg equilibrium (P-value < 10^{-8}). To account for differences in sequencing depth between cases and controls, variant sites were excluded if they had call rate < 0.97 in the full sample, or in the cases, the general controls, or controls from the Cardiff AD project.

Variant annotation

Variants were annotated with their most severe consequence across transcripts using the Ensembl Variant Effect Predictor (version 96[20]). PTVs were defined as nonsense, splice-site or frameshift variants. Missense variants were annotated using the MPC score, which stands for 'missense badness, Polyphen-2 and constraint'[21]. This is a single score that combines information from orthogonal deleteriousness metrics.

Analysis of SCHEMA rare coding variant data

We re-analysed RCVs from 24,248 cases and 97,322 controls in the SCHEMA study using variant-level data obtained from the SCHEMA Browser (https://schema.broadinstitute.org/downloads). SCHEMA case-control variants are derived from 12 sample collections and 46,885 external gnomAD controls, which the SCHEMA study stratified into 11 independent groups based on ancestry and exome capture platform (Supplementary Table 2)[17]. To control for sequencing technology and ancestry in the SCHEMA sample, we analysed RCVs in the SCHEMA cases and controls using Cochran-Mantel-Haenszel (CMH) tests with continuity correction, with separate contingency tables for the 11 SCHEMA strata. Per-gene CMH P-values for synonymous variants (MAC $\leq$ 5, which is the same MAC used in the SCHEMA study) followed the expected null distribution (Supplementary Figure 5), suggesting our CMH tests are well-controlled for stratification within the SCHEMA case-control sample.

Stratum	SCHEMA Cases	SCHEMA controls	gnomAD controls
EUR (Exomes, Nextera)	8874	19074	23561
EUR (Exomes, non-Nextera)	7277	11187	0
AMR (Exomes, Nextera)	1388	3146	12008
FIN (Exomes, non-Nextera)	944	7984	3542
EAS (Exomes, non-Nextera)	1730	1607	6806
AFR (Whole Genomes)	2245	1170	420
ASJ (Exomes, Nextera)	869	2415	548
EST (Whole Genomes)	261	2281	0
FIN (Whole Genomes)	423	655	0
AFR (Exomes, non-Nextera)	127	765	0
SAS (Exomes, non-Nextera)	110	153	0

Supplementary Table 2: SCHEMA sample counts across 11 strata defined by ancestry and exome capture platform. Strata description and sample numbers were taken from Singh et al. (2022)[17].

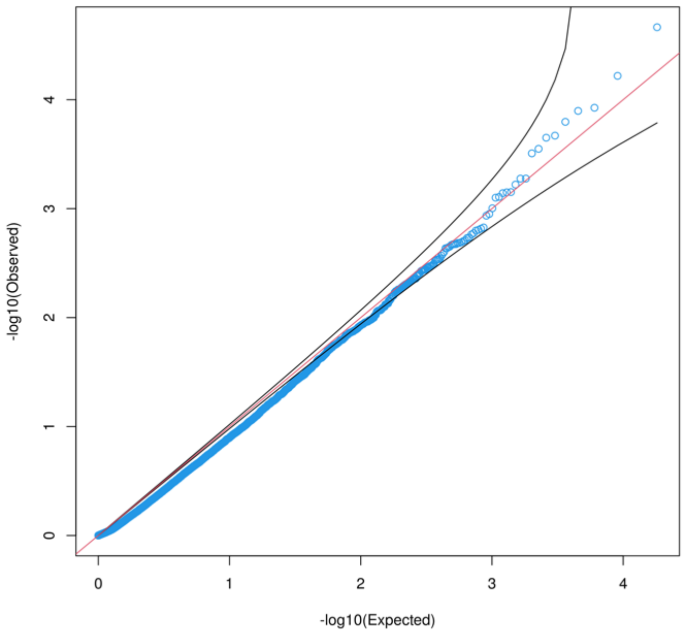

Supplementary Figure 5. QQ plot showing per-gene synonymous variant P-values generated using two-sided Cochran-Mantel-Haenszel tests over the 11 SCHEMA case-control strata.

Gene set analysis in the new case-control sample

Gene set enrichment analysis was performed in the new case-control sample using Firth's penalised logistic regression (logistf function in R (v4.2.3)), where case-control status was regressed on the number of rare coding variants in a given gene set, controlling for the first ten PCs derived from common variants in the exome-sequencing data, sex, and the exome-wide burden of rare coding variants.

We compared effect sizes between independent gene-set enrichment tests using the following z-test equation:

$$\frac{(\beta_1 - \beta_2)}{\sqrt{(\sigma_1)^2 + (\sigma_2)^2}}$$

Where β_1 represents the beta and σ_1 the standard error for burden of variants for gene-set 1, and β_2 represents the beta and σ_2 the standard error for burden of variants in gene-set 2.

Analysis of previously implicated genes in the new case-control sample

In our new case-control sample, we performed a single-gene analysis for 12 genes previously associated with RCVs in schizophrenia at exome-wide significance. We restricted this analysis to variants with a MAC $\leq$ 5 in the new case-control sample and a MAC $\leq$ 5 in the control subsample of gnomAD[22], which closely aligns with the MAC used in the SCHEMA study. For each gene, we tested the class of variant most strongly associated with schizophrenia in our re-analysis of SCHEMA case-control RCVs (described above). Since this analysis only involved our new sample, which is homogenous in terms of genetically inferred ancestry and sequencing platform, association statistics were generated for autosomal and pseudoautosomal genes using one-sided Fisher's Exact tests. For non-pseudoautosomal genes on the X chromosome, association statistics were generated using one-sided CMH tests, with separate contingency tables for males and females.

Supplementary Note 1

Relationship between sequencing depth and burden of singleton coding variants

In the new sample, sequencing coverage was greater in controls (mean genotype coverage = 34.2x) than in cases (mean genotype coverage = 26.0x). To evaluate whether this coverage difference impacts our case-control RCV enrichment analysis, we first examined the relationship between sequencing depth and the rate of singleton (minor allele count = 1 and absent in gnomAD controls[23]) coding variants in the new sample. We did this using linear regression models, where the number of singleton coding variants in LoF-tolerant genes is the dependent variable, and the per-sample mean genotype depth is the independent variable, controlling for 10 PCs and sex. We found that the collective burden of synonymous variants, missense variants and PTVs was positively correlated with mean genotype depth (Supplementary Table 3). Moreover, when tested independently, both synonymous variants and missense variants were associated with mean genotype depth (Supplementary Table 3).

Variant class	Mean genotype depth	
	Beta	P-value
Synonymous variants + missense variants + PTVs	0.026	0.0034
Synonymous	0.011	0.0068
Missense	0.014	0.044
PTVs	0.0016	0.49

Supplementary Table 3. Relationship between sequencing depth and burden of singleton rare coding variants in the new case-control sample. Beta coefficients and P-values are derived from two-sided linear regression models, where the number of singleton coding variants in LoF-tolerant genes is the dependent variable, and the per-sample mean genotype depth is the independent variable, controlling for 10 PCs and sex. P-values are two sided.

We further explored the relationship between sequencing coverage and burden of singleton coding variants in a dataset where the new controls were matched for sequencing coverage with the new cases. This dataset was generated using BAM files from wave 1 sequencing of the new controls, which is matched in sequence coverage (mean genotype depth = 27.5x) with cases (mean genotype depth = 27.7x). After applying the same QC procedures described in Supplementary Methods above, the coverage matched dataset consisted of 4,757 cases and 4,881 controls; we note there are fewer controls here compared with the original analysis because wave 1 sequencing is only available for 82% of the Cardiff AD control sample. The 107 additional cases in the coverage matched dataset are due to differences in QC thresholds that are determined by the data. In the coverage matched dataset, the burden of singleton variants in non-constrained genes did not differ between cases and controls for any class of mutation tested (Supplementary Table 4). Moreover, the excess in cases of singleton PTVs and missense variants with MPC scores > 3 was greater in this sensitivity analysis when compared with the original analysis. This suggests that the higher control sequence coverage in the original dataset leads to conservative estimates of schizophrenia rare variant enrichment, rather than false positive discovery.

**Analysis**	**Variant class**	**Original dataset**		**Coverage matched dataset**	
		**OR (95% CI)**	**P**	**OR (95% CI)**	**P**
**Constrained genes (n = 3,051)**	PTV	1.17 (1.05 – 1.30)	0.0034	1.19 (1.06 – 1.35)	0.0040
	MPC3	1.63 (1.11 – 2.40)	0.012	1.83 (1.20 – 2.80)	0.0052
	MPC 2-3	1.06 (0.97 – 1.17)	0.21	1.02 (0.92 – 1.14)	0.68
	Synonymous	0.93 (0.89 - 0.96)	2.5×10^{-5}	0.93 (0.89 - 0.97)	2.2×10^{-4}
**Non-constrained genes (n = 15,605)**	PTV	1.0 (0.97 – 1.04)	0.85	1.0 (0.96 – 1.04)	0.99
	MPC3	0.92 (0.59 – 1.41)	0.69	0.72 (0.43 – 1.18)	0.20
	MPC 2-3	1.04 (0.93 – 1.16)	0.49	1.03 (0.90 – 1.17)	0.67
	Synonymous	0.97 (0.95 - 0.99)	0.012	0.99 (0.97 - 1.01)	0.36

Supplementary Table 4. Gene-set analysis of constrained and non-constrained genes in the original dataset and in the coverage matched dataset. Odds ratios (OR) and P-values are derived from two-sided Firth's logistic regression models. Tests of singleton

PTVs and missense variants in constrained genes covary for 10 principal components, sex and total number of singleton variants exome-wide. All other tests covary for 10 principal components and sex.

Supplementary Note 2

Summary of novel schizophrenia FDR < 5% genes

SLC6A1

SLC6A1 encodes the gamma-aminobutyric acid (GABA) transporter GAT1, which is expressed in neurons and mediates uptake of GABA from the synaptic cleft of inhibitory synapses. Recently published *in vitro* GABA uptake assay data suggests schizophrenia *SLC6A1* missense variants confer loss-of-function effects on GAT-1 protein leading to reduced GABA uptake[24]. A previous schizophrenia CNV study also found genes related to the $GABA_A$ receptor complex gene set are enriched for both deletions and duplications in cases compared to controls[25].

Missense variants and PTVs in *SLC6A1* also confer risk to other psychiatric and developmental disorders. A set of *SLC6A1*-related disorder (SRD) patients has been identified from clinical cohorts of ID and childhood epilepsy; SRD is typically characterised by myoclonic-atonic epilepsy, mild or moderate ID, and autistic features[26–28]. *SLC6A1* is also significantly implicated in rare variant association studies of DD/ID and autism[29,30].

PCLO

PCLO codes for Piccolo, a component of the presynaptic cytoskeletal matrix at glutamatergic and GABAergic synapses[31]. Piccolo is thought to act as a scaffold protein promoting formation of the synapse and coordination of synaptic vesicles at the active zone where neurotransmitters are released[32]. Complete loss-of-function of *PCLO* leads to pontocerebellar hypoplasia type III in humans[33] and in rat models[34], which in humans encompasses global developmental delay and seizures. In the PGC3SEQ targeted sequencing meta-analysis, *PCLO* was implicated as a shared risk gene between schizophrenia and ASD[35].

ZMYND11

ZMYND11 encodes a chromatin remodelling protein which recognises H3.3K36me3 marks at actively transcribed genes and acts to inhibit transcriptional elongation[36]. The H3.3 histone variant has been shown to accumulate in the neuronal genome and to be particularly relevant for expression of synaptic genes[37]. *ZMYND11* has also been found to contribute to regulation of neuronal differentiation[38]. Mutations in *ZMYND11* are associated with syndromic intellectual disability, encompassing developmental delay, epilepsy, and features of ASD and ADHD[39,40]. The majority of identified mutations are protein-truncating, with a small number of missense and other mutations; missense variants have also been identified in two patients with unusually severe phenotypes, which were suspected to lead to gain-of-function effects[41,42].

BSCL2

BSCL2 encodes seipin, an endoplasmic reticulum (ER)-localised protein which mediates formation of lipid droplets as an energy store within the cell[43]. PTVs in *BSCL2* are associated with severe lipodystrophy, encompassing diabetes and intellectual disability, and gain-of-function variants are associated with a spectrum of neuropathic conditions including distal hereditary motor neuropathy, Silver syndrome, and spastic paraplegia[44]. Splice variants in *BSCL2* have also been suggested to cause epileptic encephalopathy[45] and ASD with parkinsonism[46].

KLC1

KLC1 codes for a light chain subunit of kinesin, a tetrameric protein complex responsible for intracellular transport along the cytoskeleton. Two heavy chains function as motors, while two light chains act as adaptors binding cargo to be transported[47]. In neurons, *KLC1* has been shown to play a role in vesicle transport through its interaction with Calsyntenin-2[48], and knockdown of *KLC1* in a human cell line was found to impair neuronal differentiation[49]. In addition to implication of *KLC1* in schizophrenia by the largest GWAS to date[50], summary-based Mendelian randomization[51] and transcription-wide association[52] analyses indicate that common

schizophrenia-associated variants at the locus are associated with reduced expression of *KLC1* protein-coding transcripts in the prenatal brain.

CGREF1

CGREF1 encodes a secretory protein which has previously been identified as an autism risk gene[30]. Although there is evidence that it plays a role in cell proliferation[53], single nuclei RNA profiling indicate predominant *CGREF1* expression in post-mitotic neurons, with undetected expression in proliferating progenitor populations (see section 4c below).

Supplementary Note 3

Expression profiles of genes implicated in schizophrenia at exome-wide significance and FDR < 5%

Expression in adult human tissue

We used the FUMA platform (https://fuma.ctglab.nl)[54] to assess the normal expression of the 8 genes implicated in schizophrenia by the current study at exome-wide significance and FDR < 5% across 54 adult human tissues (Supplementary Figure 6). All 8 genes displayed detectable expression in at least one adult brain region. *BSCL2*, *KLC1* and *ZMYND11* are broadly expressed across adult tissues, whereas *CGREF1*, *PCLO* and *SLC6A1* expression is largely restricted to the brain.

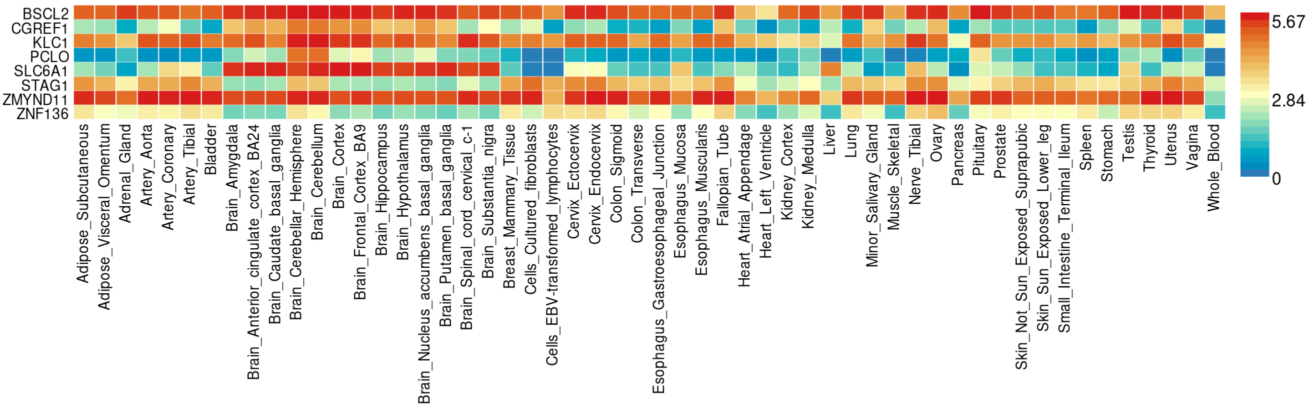

Supplementary Figure 6. RNA expression profile of genes implicated in schizophrenia in this study (exome-wide significant and FDR < 5%) across adult human tissues. Figure generated using the FUMA platform[54] based on bulk RNA sequencing of 54 adult tissues by the GTEx Consortium[55] and average expression per tissue (log2 transformed).

Developmental expression in 6 human brain regions

We used the Human Brain Transcriptome database (https://hbatlas.org/)[56] to assess the developmental RNA expression of the 8 genes implicated in schizophrenia by the

current study at exome-wide significance and FDR < 5% in 6 human brain regions from 5 weeks of gestation to 82 years of age (Supplementary Figure 7). *STAG1* and *ZNF136* display higher pre-natal expression in all assayed brain regions, whereas *CGREF1* and *SLC6A1* exhibit higher post-natal expression.

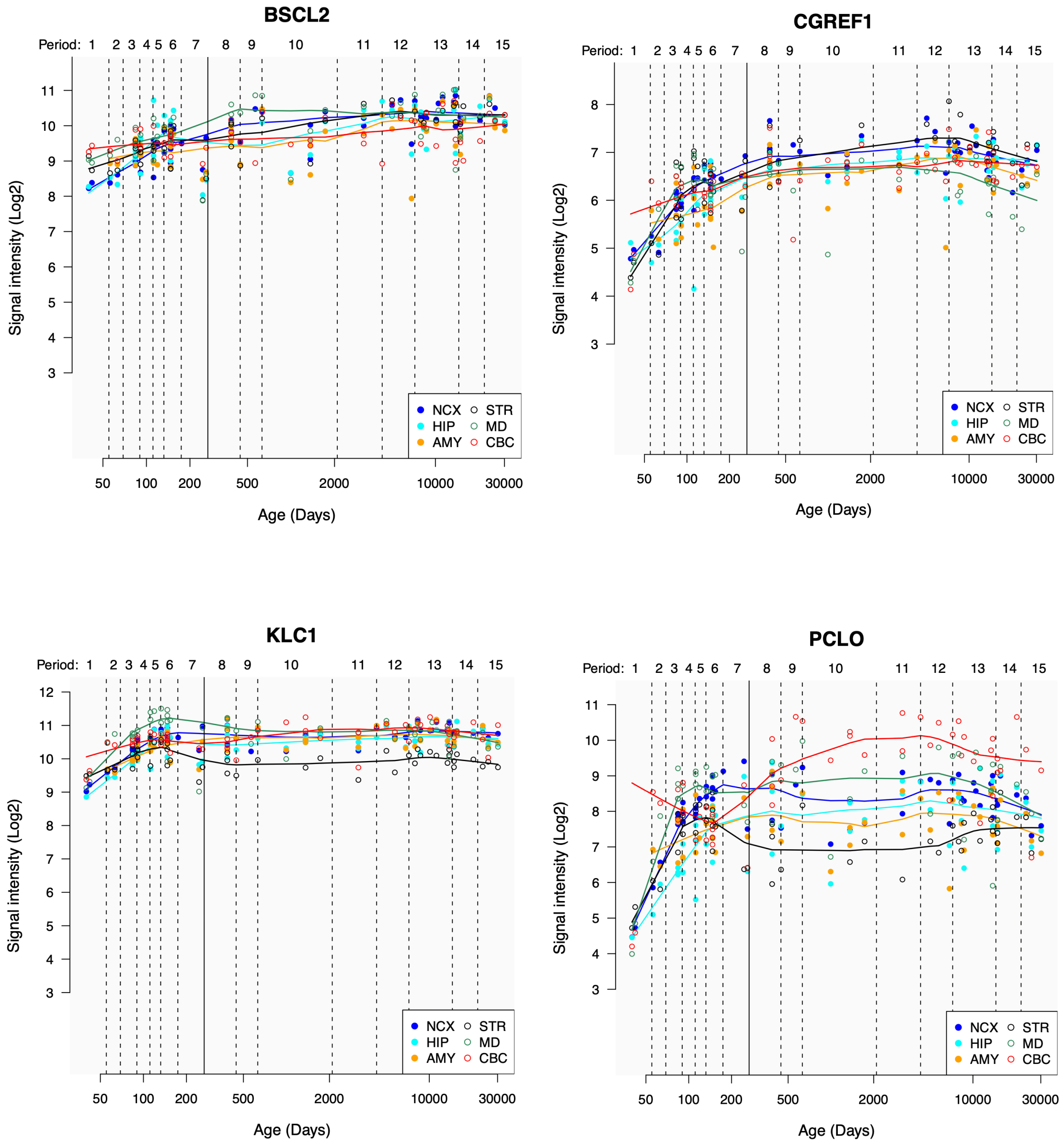

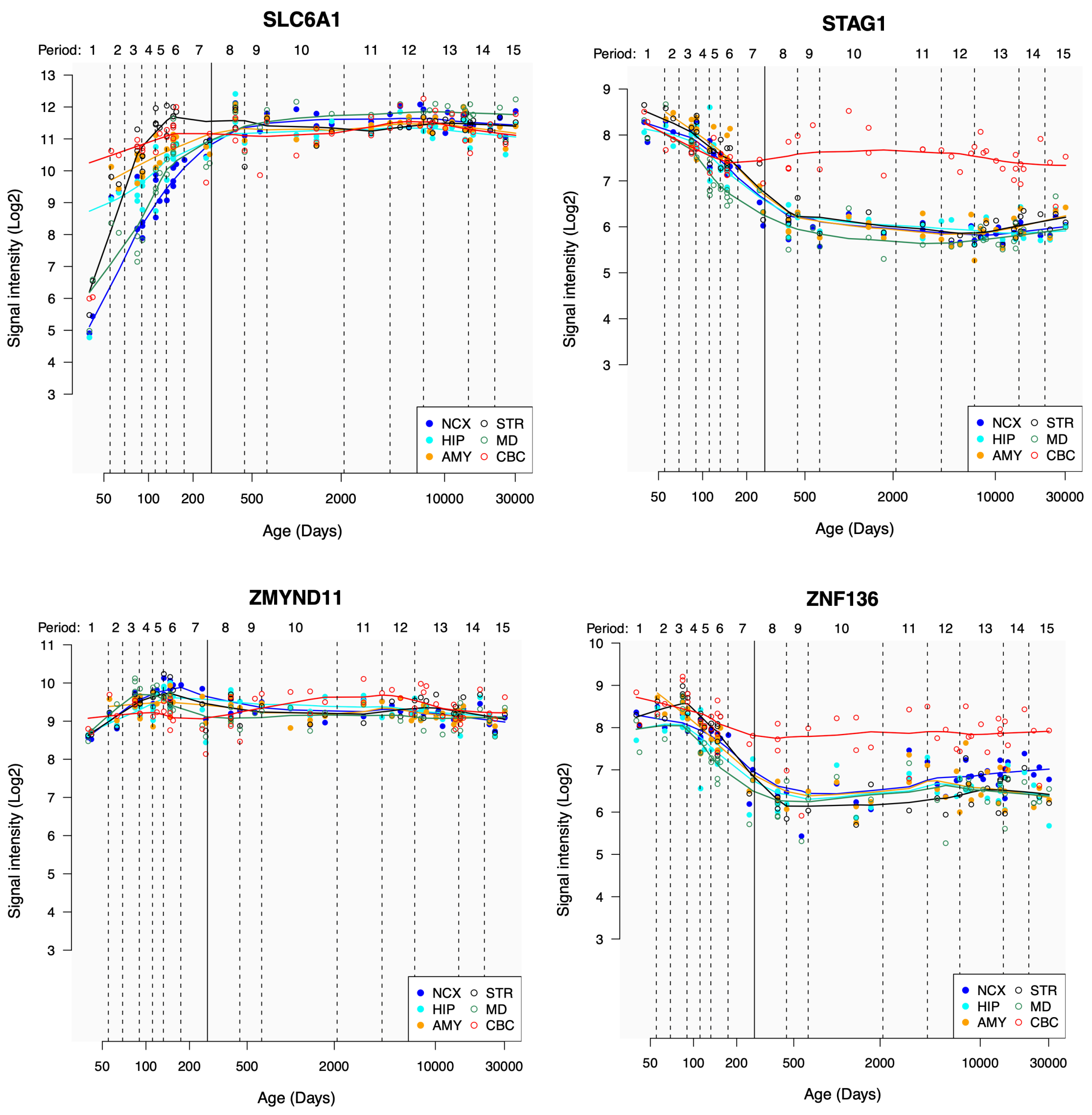

Supplementary Figure 7. Developmental expression of genes implicated in schizophrenia in this study in 6 regions of the adult brain. Figures generated through the Human Brain Transcriptome database (https://hbatlas.org/) based on microarray profiling of the neocortex (NCX), striatum (STR), hippocampus (HIP), mediodorsal nucleus of the thalamus (MD), amydgala (AMY) and cerebellar cortex (CBC) in human samples ranging from 5 weeks of gestation to 82 years of age[56]. Period 1 = 4-8 postconception weeks (PCW); Period 2 = 8-10 PCW; Period 3 = 10-13 PCW; Period 4 = 13-16 PCW; Period 5 = 16-19 PCW; Period 6 = 19-24 PCW; Period 7 = 24-38 PCW; Period 8 = birth-6 months; Period 9 = 6-12 months; Period 10 = 1-6 years; Period 11 = 6-12 years; Period 12 = 12-20 years; Period 13 = 20-40 years; Period 14 = 40-60 years; Period 15 = 60-82 years.

Cellular expression in fetal and adult human frontal cortex

To assess the cellular expression of the 8 genes implicated in schizophrenia by the current study at exome-wide significance and FDR < 5%, we analysed single nuclei RNA sequencing data from the fetal[57] and adult[58] human frontal cortex (Supplementary Figures 8 and 9). Several genes are expressed across all neural cell populations; notable exceptions are *SLC6A1*, which is largely restricted to GABAergic neurons of the post-natal brain, and *CGREF1*, where expression is limited to post-natal glutamatergic and GABAergic neurons.

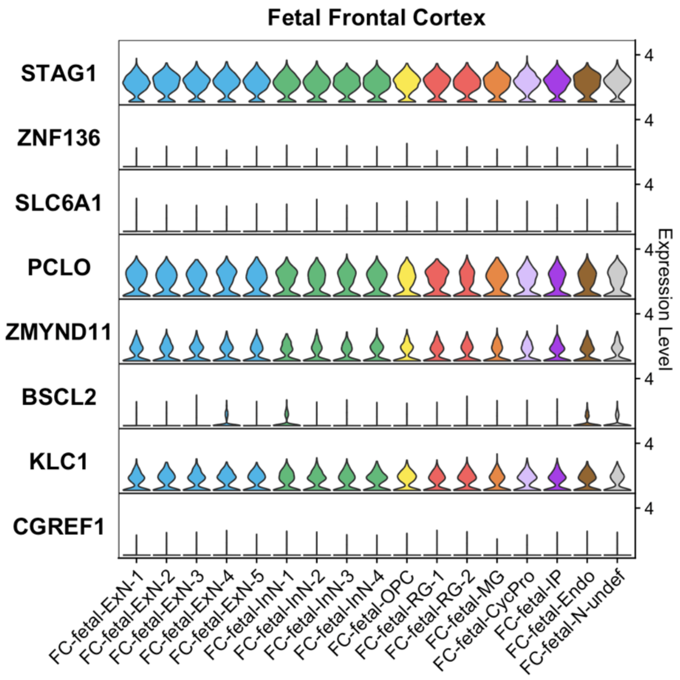

Supplementary Figure 8. Expression of implicated genes in the human fetal frontal cortex. Data generated through single nuclei RNA sequencing of the 14-15 post-conception week human fetal brain[57] and visualized as violin plots based on normalized, log-transformed expression values using[59]. Cell populations are labelled according to marker genes[57]. FC = frontal cortex; ExN = excitatory (glutamatergic) neurons; InN = inhibitory (GABAergic) neurons; OPC = oligodendrocyte precursor cell; RG = radial glia; MG = microglia; CycPro = *MKI67* expressing cycling progenitor cells; IP = intermediate progenitors; Endo = endothelial cell; N-undef = neuron of undefined class.

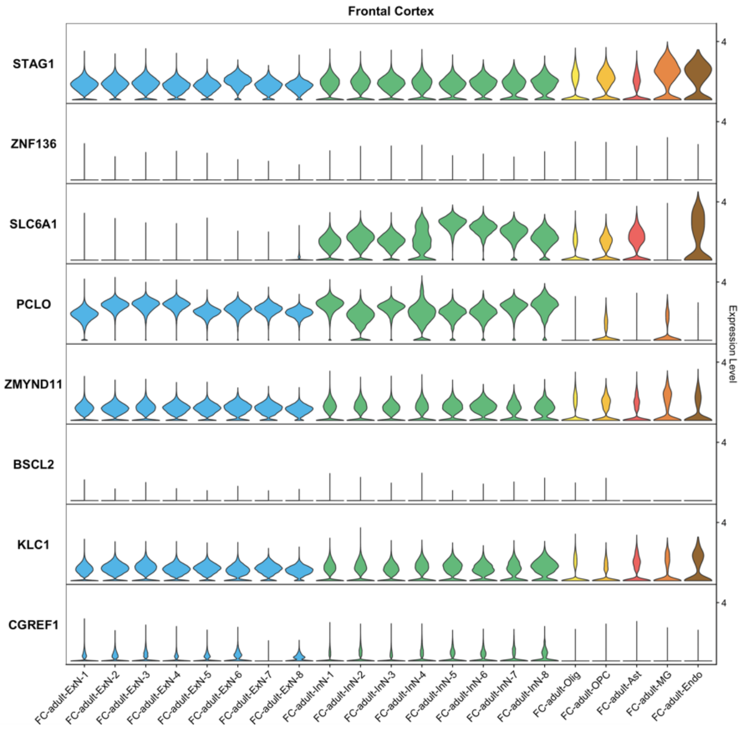

Supplementary Figure 9. Expression of implicated genes in the adult human frontal cortex. Data generated through single nuclei RNA sequencing of the adult human brain[58] and visualized as violin plots based on normalized, log-transformed expression values using[59]. Cell populations are labelled according to marker genes. FC = frontal cortex; ExN = excitatory (glutamatergic) neurons; InN = inhibitory (GABAergic) neurons; Olig = oligodendrocyte; OPC = oligodendrocyte precursor cell; Ast = astrocyte; MG = microglia; Endo = endothelial cell.

Supplementary Tables 5-11

Gene set	Variant class	N variants (rate)		OR (95% CI)	P
		Cases	Controls		
Constrained genes (n = 3,051)	PTV	869 (0.19)	970 (0.17)	1.17 (1.05-1.30)	3.4×10^{-3}
	MPC > 3	70 (0.015)	61 (0.011)	1.63 (1.11-2.40)	1.2×10^{-2}
	MPC 2-3	1068 (0.23)	1346 (0.24)	1.04 (0.94-1.14)	0.45
	Synonymous	6764 (1.5)	9990 (1.7)	0.93 (0.89-0.96)	2.5×10^{-5}

Supplementary Table 5: Gene-set analysis of singleton coding variants in constrained genes in the new case-control sample. P values and odds ratios (OR) were derived from Firth's logistic regression models (Supplementary Methods). P values are two-sided and uncorrected for multiple comparisons. Constrained genes are defined as those with pLi scores ≥ 0.9 in gnomAD[22]. PTV = protein-truncating variants; MPC = 'missense badness, Polyphen-2 and constraint' score; pLi = probability of being loss of function intolerant.

CNV	CNV coordinates		
	Chr	Start	End
**1q21.1 del**	1	146527987	147394444
**1q21.1 dup**	1	146527987	147394444
**NRXN1 del**	2	50145643	51259674
**3q29 del**	3	195720167	197354826
**WBS dup**	7	72744915	74142892
**PWS/AS dup**	15	22805313	28390339
**15q11.2 BP1-BP2 del**	15	22805313	23094530
**15q13.3 del**	15	31080645	32462776
**16p13.11 dup**	16	15511655	16293689
**16p12.1 del**	16	21950135	22431889
**16p11.2 distal del**	16	28823196	29046783
**16p11.2 dup**	16	29650840	30200773
**22q11.2 (DiGeorge/VCFS syndrome) del**	22	19037332	21466726

Supplementary Table 6. List of known schizophrenia associated CNVs. Associated CNVs taken from Rees *et al* 2016[60] and Marshall *et al* 2017[59]. CNV coordinates are in build37/hg19 and map to the critical regions as defined in Rees *et al* 2016[60]. CNV = copy number variant; del = deletion; dup = duplication.

CNV locus (N gene tests)	Single gene RCV enrichment statistics				
	Gene symbol	Gene locus	Variant type	P-value (uncorrected)	Odds ratio
NRXN1 del (3)	*NRXN1*	2:50145643-51259674	PTV	0.000291*	5.9 (2.2, 15.8)
22q11.2 del (70)	*C22orf39*	22:19338891-19435755	PTV	0.00384	21.1 (2, 222.9)
3q29 del (38)	*UBXN7*	3:196074533-196159345	PTV + MPC >3	0.00492	15.8 (2.2, 115.7)
WBS dup (41)	*LIMK1*	7:73497263-73536855	PTV	0.0146	Inf (NA, NA)
16p11.2 dup (46)	*TAOK2*	16:29984962-30003582	PTV + MPC >2	0.0148	2.1 (1.2, 3.6)

Supplementary Table 7. Rare coding variant enriched genes in schizophrenia CNV loci. Genes are shown if they were enriched in the case-control-*de novo* meta-analysis (described in Methods) for rare coding variants with an uncorrected P-value < 0.05 and overlapped a known schizophrenia CNV locus (listed in Supplementary Table 5). "N gene tests" gives the total number of tests for a given CNV locus (Σ genes $\times$ mutation classes tested). * indicates P-values that survive Bonferroni correction for the number of genes tested in the given CNV locus. P values are two-sided and uncorrected for multiple comparisons. RCV = rare coding variant; CI = confidence interval; CNV = copy number variant; del = deletion; dup = duplication; Inf = infinity. Gene locus coordinates are in build 37/hg19.

Gene set	Variant class	N variants (rate)		OR (95% CI)	P
		Cases	Controls		
Published exome-wide significant genes (n = 12)	PTV	25 (0.0054)	7 (0.0012)	4.97 (2.05-13.31)	2.7×10^{-4}
	MPC >3	5 (0.0011)	7 (0.0012)	0.90 (0.23-3.13)	0.87
	MPC 2-3	18 (0.0039)	21 (0.0037)	1.24 (0.59-2.59)	0.57
	Synonymous	223 (0.048)	295 (0.052)	0.94 (0.76-1.15)	0.53
Published FDR < 5% genes (n = 20)	PTV	21 (0.0045)	16 (0.0028)	2.48 (1.19-5.22)	1.6×10^{-2}
	MPC >3	0 (0)	0 (0)	NA	NA
	MPC 2-3	21 (0.0045)	28 (0.0049)	1.00 (0.52-1.90)	0.99
	Synonymous	230 (0.049)	293 (0.051)	0.96 (0.79-1.18)	0.72

Supplementary Table 8. Gene-set analysis of previously implicated genes in the new case-control sample. Variants are restricted to those with a minor allele count ≤ 5 in the new sample and ≤ 5 in gnomAD controls. Odds ratios (OR) and P values were derived from Firth's logistic regression models (Supplementary Methods). P values are two-sided and uncorrected for multiple comparisons. Published exome-wide significant genes were derived from[17,35]. Additional published FDR < 5% genes were derived from[17]. PTV = protein-truncating variants; MPC = 'missense badness, Polyphen-2 and constraint' score.

Gene set	Gene-set enrichment			Z-test P-value
	beta	SE	P	
12 previously implicated exome-wide significant genes	1.60	0.468	2.7×10^{-4}	8.1×10^{-4}
Independent set of constrained genes	0.121	0.0447	0.0068	
20 additional previously implicated genes at FDR < 5%	0.908	0.373	0.016	0.018
Independent set of constrained genes	0.121	0.0447	0.0068	

Supplementary Table 9. Comparison of effect sizes for PTVs in previously implicated genes and constrained genes. Variants are restricted to PTVs with a minor allele count ≤ 5 in the new sample and ≤ 5 in gnomAD controls. Beta values, standard errors (SE) and P-values were derived from Firth's logistic regression models in the new case-control sample. P values are two-sided and uncorrected for multiple comparisons. See Supplementary Methods for a description of the Z-test used to compare gene-set enrichment statistics.

Gene symbol	Variant class	New sample			
		Case variants	Control variants	OR (95% CI)	P-val
SETD1A	PTV + MPC>2	8	2	4.9 (1.0-47.6)	0.027
XPO7	PTV + MPC>3	4	0	Inf (0.8-Inf)	0.040
HERC1	PTV	4	1	4.9 (0.5-242.2)	0.13
TRIO	PTV	0	1	0.0 (0.0-47.9)	1.00
SP4	PTV + MPC>3	7	5	1.7 (0.5-6.9)	0.26
GRIN2A	PTV + MPC>2	3	2	1.8 (0.2-22.1)	0.40
CACNA1G	PTV + MPC>3	0	4	0.0 (0.0-1.9)	1.00
CUL1	PTV + MPC>3	0	1	0.0 (0.0-47.9)	1.00
AKAP11	PTV	3	0	Inf (0.5-Inf)	0.090
SRRM2	PTV	5	0	Inf (1.1-Inf)	0.018
GRIA3	PTV + MPC>3	0	0	0.0 (0.0-Inf)	1.00
RB1CC1	PTV	2	1	2.5 (0.1-145.1)	0.42

Supplementary Table 10. Analysis of previously reported exome-wide significant genes in the new case-control data. Variant counts, odds ratios (ORs) and P-values correspond to the variant class shown. Variant classes and ordering correspond to SCHEMA re-analysis P-values (Supplementary Data 2). P-values for autosomal genes were calculated from 1-sided Fisher Exact tests. The P-value for *GRIA3*, which is on the X chromosome, was calculated from a one-sided Cochran-Manel-Haenzel test. All P values are uncorrected for multiple comparisons. ORs were calculated from 2-sided Fisher Exact and Cochran-Manel-Haenzel tests. CI = confidence interval.

Gene symbol	Variant class	Case-control-de novo variant meta-analysis	
		P-val	Q-val
STAG1	PTV + MPC >2	**1.5×10^{-7}**	4.6×10^{-4}
ZNF136	PTV	**1.5×10^{-6}**	0.0031
SLC6A1	MPC >2	3.6×10^{-6}	0.0046
KLC1	MPC >2	1.1×10^{-5}	0.012
PCLO	PTV	2.4×10^{-5}	0.024
ZMYND11	PTV	2.5×10^{-5}	0.024
BSCL2	PTV	5.3×10^{-5}	0.043
CGREF1	PTV	8.3×10^{-5}	0.061

Supplementary Table 11. Sensitivity analysis of novel exome-wide significant and FDR < 5% genes. P values were calculated using the case-control-*de novo* meta-analysis described in the Methods, excluding individuals with Alzheimer's disease excluded from the new control sample. P values are two-sided and uncorrected for multiple comparisons, with bold text indicating those exceeding Bonferroni significance ($P < 1.63 \times 10^{-6}$). Q-values show adjusted P values using the false discovery rate approach. In this sensitivity analysis, both *STAG1* and *ZNF136* remained associated at exome-wide significance, and among the 6 additional FDR < 5% significant novel genes, only *CGREF1* is no longer significant at FDR < 5% (Q-value = 0.056). PTV = protein-truncating variant; MPC = 'missense badness, Polyphen-2 and constraint' scores for missense variants.

Supplementary Figure 10

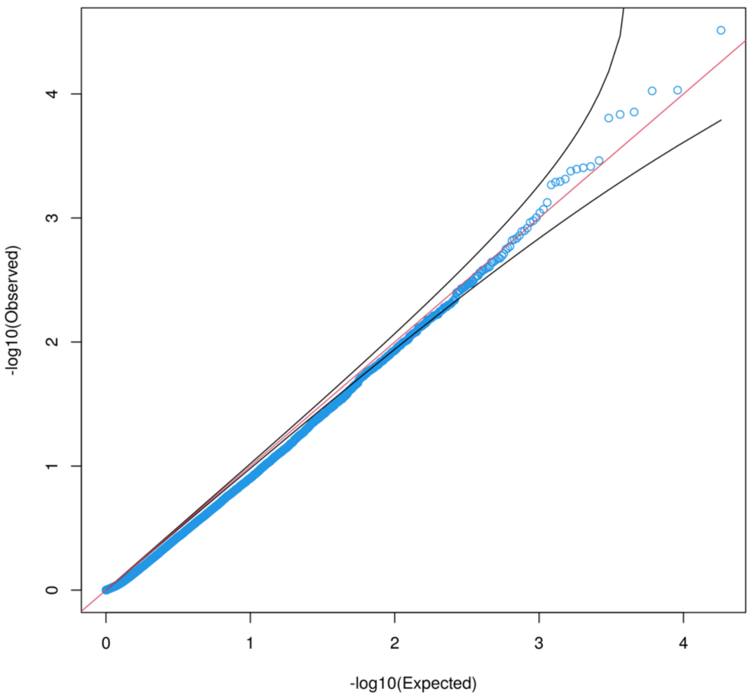

Supplementary Figure 10. QQ plot for synonymous variant P-values in the combined case-control meta-analysis (SCHEMA + new sample). P-values are 2-sided and were generated using Cochran-Mantel-Haenszel tests with 12 contingency tables for autosomal and pseudoautosomal genes and 13 contingency tables for non-pseudoautosomal genes (full approach described in Methods).

Supplementary References

1. Pardiñas, A. F. *et al.* Common schizophrenia alleles are enriched in mutation-intolerant genes and in regions under strong background selection. *Nature genetics* **50**, 381–389 (2018).
2. Lynham, A. J. *et al.* Examining cognition across the bipolar/schizophrenia diagnostic spectrum. *J Psychiatry Neurosci* **43**, 245–253 (2018).
3. Creeth, H. D. J. *et al.* Ultrarare Coding Variants and Cognitive Function in Schizophrenia. *JAMA Psychiatry* **79**, 963–970 (2022).
4. Williams, N. M. *et al.* Variation at the DAOA/G30 Locus Influences Susceptibility to Major Mood Episodes but Not Psychosis in Schizophrenia and Bipolar Disorder. *Archives of General Psychiatry* **63**, 366–373 (2006).
5. Cardno, A. G. *et al.* Dimensions of psychosis in affected sibling pairs. *Schizophr Bull* **25**, 841–850 (1999).
6. *Diagnostic and Statistical Manual of Mental Disorders: DSM-IV ; Includes ICD-9-CM Codes Effective 1. Oct. 96*. (Washington, DC, 1998).
7. World Health Organization. *The ICD-10 Classification of Mental and Behavioural Disorders : Clinical Descriptions and Diagnostic Guidelines*. https://apps.who.int/iris/handle/10665/37958 (1992).
8. Power, C. & Elliott, J. Cohort profile: 1958 British birth cohort (National Child Development Study). *Int J Epidemiol* **35**, 34–41 (2006).
9. Wellcome Trust Case Control Consortium. Genome-wide association study of 14,000 cases of seven common diseases and 3,000 shared controls. *Nature* **447**, 661–678 (2007).

10. Lynham, A. J. *et al.* DRAGON-Data: a platform and protocol for integrating genomic and phenotypic data across large psychiatric cohorts. *BJPsych Open* **9**, e32 (2023).
11. Moskvina, V., Holmans, P., Schmidt, K. M. & Craddock, N. Design of Case-controls Studies with Unscreened Controls. *Annals of Human Genetics* **69**, 566–576 (2005).
12. Ohi, K., Fujikane, D. & Shioiri, T. Genetic overlap between schizophrenia spectrum disorders and Alzheimer's disease: Current evidence and future directions - An integrative review. *Neurosci Biobehav Rev* **167**, 105900 (2024).
13. The Brainstorm Consortium *et al.* Analysis of shared heritability in common disorders of the brain. *Science* **360**, eaap8757 (2018).
14. Wingo, T. S. *et al.* Shared mechanisms across the major psychiatric and neurodegenerative diseases. *Nat Commun* **13**, 4314 (2022).
15. Pedersen, B. S. & Quinlan, A. R. Who's Who? Detecting and Resolving Sample Anomalies in Human DNA Sequencing Studies with Peddy. *The American Journal of Human Genetics* **100**, 406–413 (2017).
16. Hail Team. Hail 0.2. https://github.com/hail-is/hail. (2023).
17. Singh, T. *et al.* Rare coding variants in ten genes confer substantial risk for schizophrenia. *Nature* **604**, 509–516 (2022).
18. Lu, W. *et al.* CHARR efficiently estimates contamination from DNA sequencing data. *Am J Hum Genet* **110**, 2068–2076 (2023).
19. Li, H. Toward better understanding of artifacts in variant calling from high-coverage samples. *Bioinformatics* **30**, 2843–2851 (2014).
20. McLaren, W. *et al.* The Ensembl Variant Effect Predictor. *Genome Biology* **17**, 122 (2016).
21. Samocha, K. E. *et al.* Regional missense constraint improves variant deleteriousness prediction. 148353 Preprint at https://doi.org/10.1101/148353 (2017).

22. Karczewski, K. J. *et al.* The mutational constraint spectrum quantified from variation in 141,456 humans. *Nature* **581**, 434–443 (2020).

23. Karczewski, K. J. *et al.* The mutational constraint spectrum quantified from variation in 141,456 humans. *Nature* **581**, 434–443 (2020).

24. Silva, D. B. *et al.* Haploinsufficiency underlies the neurodevelopmental consequences of *SLC6A1* variants. *The American Journal of Human Genetics* **111**, 1222–1238 (2024).

25. Pocklington, A. J. *et al.* Novel Findings from CNVs Implicate Inhibitory and Excitatory Signaling Complexes in Schizophrenia. *Neuron* **86**, 1203–1214 (2015).

26. Goodspeed, K. *et al.* Current knowledge of SLC6A1-related neurodevelopmental disorders. *Brain Communications* **2**, fcaa170 (2020).

27. Kahen, A. *et al.* Neurodevelopmental phenotypes associated with pathogenic variants in SLC6A1. *J Med Genet* **59**, 536–543 (2022).

28. Johannesen, K. M. *et al.* The phenotypic presentation of adult individuals with SLC6A1-related neurodevelopmental disorders. *Front Neurosci* **17**, 1216653 (2023).

29. Kaplanis, J. *et al.* Evidence for 28 genetic disorders discovered by combining healthcare and research data. *Nature* **586**, 757–762 (2020).

30. Fu, J. M. *et al.* Rare coding variation provides insight into the genetic architecture and phenotypic context of autism. *Nat Genet* **54**, 1320–1331 (2022).

31. Fenster, S. D. *et al.* Piccolo, a Presynaptic Zinc Finger Protein Structurally Related to Bassoon. *Neuron* **25**, 203–214 (2000).

32. Mukherjee, K. *et al.* Piccolo and bassoon maintain synaptic vesicle clustering without directly participating in vesicle exocytosis. *Proc Natl Acad Sci U S A* **107**, 6504–6509 (2010).

33. Ahmed, M. Y. *et al.* Loss of PCLO function underlies pontocerebellar hypoplasia type III. *Neurology* **84**, 1745–1750 (2015).

34. Falck, J. *et al.* Loss of Piccolo Function in Rats Induces Cerebellar Network Dysfunction and Pontocerebellar Hypoplasia Type 3-like Phenotypes. *J Neurosci* **40**, 2943–2959 (2020).

35. Liu, D. *et al.* Schizophrenia risk conferred by rare protein-truncating variants is conserved across diverse human populations. *Nat Genet* **55**, 369–376 (2023).

36. Wen, H. *et al.* ZMYND11 links histone H3.3K36me3 to transcription elongation and tumour suppression. *Nature* **508**, 263–268 (2014).

37. Maze, I. *et al.* Critical role of histone turnover in neuronal transcription and plasticity. *Neuron* **87**, 77–94 (2015).

38. Yu, B. *et al.* BS69 undergoes SUMO modification and plays an inhibitory role in muscle and neuronal differentiation. *Exp Cell Res* **315**, 3543–3553 (2009).

39. Yates, T. M. *et al.* ZMYND11-related syndromic intellectual disability: 16 patients delineating and expanding the phenotypic spectrum. *Human Mutation* **41**, 1042–1050 (2020).

40. Oates, S. *et al.* ZMYND11 variants are a novel cause of centrotemporal and generalised epilepsies with neurodevelopmental disorder. *Clinical Genetics* **100**, 412–429 (2021).

41. Cobben, J. M. *et al.* A de novo mutation in ZMYND11, a candidate gene for 10p15.3 deletion syndrome, is associated with syndromic intellectual disability. *Eur J Med Genet* **57**, 636–638 (2014).

42. Moskowitz, A. M. *et al.* A de novo missense mutation in ZMYND11 is associated with global developmental delay, seizures, and hypotonia. *Cold Spring Harb Mol Case Stud* **2**, a000851 (2016).

43. Salo, V. T. Seipin-still a mysterious protein? *Front Cell Dev Biol* **11**, 1112954 (2023).
44. Ito, D. & Suzuki, N. Seipinopathy: a novel endoplasmic reticulum stress-associated disease. *Brain* **132**, 8–15 (2009).
45. Fernández-Marmiesse, A. *et al.* A *de novo* heterozygous missense *BSCL2* variant in 2 siblings with intractable developmental and epileptic encephalopathy. *Seizure* **71**, 161–165 (2019).
46. Poisson, A. *et al.* Regressive Autism Spectrum Disorder Expands the Phenotype of BSCL2/Seipin-Associated Neurodegeneration. *Biological Psychiatry* **85**, e17–e19 (2019).
47. Woźniak, M. J. & Allan, V. J. Cargo selection by specific kinesin light chain 1 isoforms. *The EMBO Journal* **25**, 5457–5468 (2006).
48. Vagnoni, A., Rodriguez, L., Manser, C., De Vos, K. J. & Miller, C. C. J. Phosphorylation of kinesin light chain 1 at serine 460 modulates binding and trafficking of calsyntenin-1. *Journal of Cell Science* **124**, 1032–1042 (2011).
49. Killian, R. L., Flippin, J. D., Herrera, C. M., Almenar-Queralt, A. & Goldstein, L. S. B. Kinesin Light Chain 1 Suppression Impairs Human Embryonic Stem Cell Neural Differentiation and Amyloid Precursor Protein Metabolism. *PLOS ONE* **7**, e29755 (2012).
50. Trubetskoy, V. *et al.* Mapping genomic loci implicates genes and synaptic biology in schizophrenia. *Nature* **604**, 502–508 (2022).
51. O'Brien, H. E. *et al.* Expression quantitative trait loci in the developing human brain and their enrichment in neuropsychiatric disorders. *Genome Biol.* **19**, 194 (2018).
52. Hall, L. S. *et al.* Cis-effects on gene expression in the human prenatal brain associated with genetic risk for neuropsychiatric disorders. *Mol Psychiatry* **26**, 2082–2088 (2021).

53. Deng, W. *et al.* The novel secretory protein CGREF1 inhibits the activation of AP-1 transcriptional activity and cell proliferation. *The International Journal of Biochemistry & Cell Biology* **65**, 32–39 (2015).

54. Watanabe, K., Taskesen, E., van Bochoven, A. & Posthuma, D. Functional mapping and annotation of genetic associations with FUMA. *Nat Commun* **8**, 1826 (2017).

55. GTEx Consortium. The GTEx Consortium atlas of genetic regulatory effects across human tissues. *Science* **369**, 1318–1330 (2020).

56. Kang, H. J. *et al.* Spatio-temporal transcriptome of the human brain. *Nature* **478**, 483–489 (2011).

57. Cameron, D. *et al.* Single-Nuclei RNA Sequencing of 5 Regions of the Human Prenatal Brain Implicates Developing Neuron Populations in Genetic Risk for Schizophrenia. *Biol Psychiatry* **93**, 157–166 (2023).

58. Siletti, K. *et al.* Transcriptomic diversity of cell types across the adult human brain. *Science* **382**, eadd7046 (2023).

59. Hao, Y. *et al.* Dictionary learning for integrative, multimodal and scalable single-cell analysis. *Nat Biotechnol* **42**, 293–304 (2024).

60. Rees, E. *et al.* Analysis of intellectual disability copy number variants for association with schizophrenia. *JAMA psychiatry* **73**, 963–969 (2016).

61. Marshall, C. R. *et al.* Contribution of copy number variants to schizophrenia from a genome-wide study of 41,321 subjects. *Nature Genetics* **49**, 27–35 (2017).
