# Supplementary material for: Whole-exome sequencing analysis identifies risk genes for schizophrenia
Source: Nat Commun. 2025 Aug 2;16:7102. doi: 10.1038/s41467-025-62429-y (PMC12318047; doi:10.1038/s41467-025-62429-y)
Supplement: Supplementary file 2 — Description of Additional Supplementary Files [file 41467_2025_62429_MOESM2_ESM.pdf]

Description of Additional Supplementary Files

Supplementary Data 1: Variants in new case-control sample that contribute to novel exome-wide significant and FDR < 5% genes. Locus coordinates are in build37. MPC = 'missense badness, Polyphen-2 and constraint' score.

Supplementary Data 2: Full gene case-control and de novo enrichment results. SCHEMA case-control variant counts are derived from our re-analysis of variant level data downloaded from the SCHEMA Browser (https://schema.broadinstitute.org/downloads).
