# Supplementary material for: Whole-exome sequencing analysis identifies risk genes for schizophrenia
Source: Nat Commun. 2025 Aug 2;16:7102. doi: 10.1038/s41467-025-62429-y (PMC12318047; doi:10.1038/s41467-025-62429-y)
Supplement: Supplementary file 5 — Reporting Summary [file 41467_2025_62429_MOESM5_ESM.pdf]

nature portfolio

Corresponding author(s): Elliott Rees

Last updated by author(s): Jul 3, 2025

Reporting Summary

Nature Portfolio wishes to improve the reproducibility of the work that we publish. This form provides structure for consistency and transparency in reporting. For further information on Nature Portfolio policies, see our Editorial Policies and the Editorial Policy Checklist.

Statistics

For all statistical analyses, confirm that the following items are present in the figure legend, table legend, main text, or Methods section.

n/a	Confirmed	
☐	☒	The exact sample size (*n*) for each experimental group/condition, given as a discrete number and unit of measurement
☐	☒	A statement on whether measurements were taken from distinct samples or whether the same sample was measured repeatedly
☐	☒	The statistical test(s) used AND whether they are one- or two-sided<br>*Only common tests should be described solely by name; describe more complex techniques in the Methods section.*
☐	☒	A description of all covariates tested
☐	☒	A description of any assumptions or corrections, such as tests of normality and adjustment for multiple comparisons
☐	☒	A full description of the statistical parameters including central tendency (e.g. means) or other basic estimates (e.g. regression coefficient) AND variation (e.g. standard deviation) or associated estimates of uncertainty (e.g. confidence intervals)
☐	☒	For null hypothesis testing, the test statistic (e.g. *F*, *t*, *r*) with confidence intervals, effect sizes, degrees of freedom and *P* value noted<br>*Give P values as exact values whenever suitable.*
☒	☐	For Bayesian analysis, information on the choice of priors and Markov chain Monte Carlo settings
☒	☐	For hierarchical and complex designs, identification of the appropriate level for tests and full reporting of outcomes
☐	☒	Estimates of effect sizes (e.g. Cohen's *d*, Pearson's *r*), indicating how they were calculated

Our web collection on statistics for biologists contains articles on many of the points above.

Software and code

Policy information about availability of computer code

Data collection	No software and code was used in data collection.
Data analysis	All software and code used for data analysis is decribed in the Methods and Supplementary Methods. Briefly, new exome sequencing data was processed in accordance with Genome Analysis ToolKit (GATK) Best Practice guidelines. The Burrow–wheeler Aligner (v0.7.15) was used to align reads to the human reference genome (GRCh37). Variants were joint-called across all samples using the GATK Haplotype Caller V3.4 and filtered using the GATK Variant Quality Score Recalibration (VQSR) tool. The analysis of VCF files was performed using Hail version 0.2.60 and R version 4.2.3. The code used to generate our gene set and single gene results are available at https://github.com/sophie-chick/SZ_gene_discovery and on Zenodo at https://doi.org/10.5281/zenodo.14865529

For manuscripts utilizing custom algorithms or software that are central to the research but not yet described in published literature, software must be made available to editors and reviewers. We strongly encourage code deposition in a community repository (e.g. GitHub). See the Nature Portfolio guidelines for submitting code & software for further information.

Data

Policy information about availability of data

All manuscripts must include a data availability statement. This statement should provide the following information, where applicable:

- Accession codes, unique identifiers, or web links for publicly available datasets
- A description of any restrictions on data availability
- For clinical datasets or third party data, please ensure that the statement adheres to our policy

The genetic variants contributing to the novel schizophrenia genes reported in the current study are presented in Supplementary Data 1. Aggregated variant counts at the gene level are provided in Supplementary Data 2. All datasets included in the current study are described in the Methods and Supplementary Methods. Individuals in the new case sample did not consent to public sharing of their raw genetic data, and we do not have ethical approval to deposit their exome-sequence data in controlled access repositories such as dbGaP or the EGA. Exome-sequence data from the new cases is only available through collaboration with the relevant PI, who can be contacted via the corresponding author (E.R). Through our collaboration with SCHEMA, exome-sequence data from the new cases have been deposited into the SCHEMA Consortium. As described in the SCHEMA paper, requests for access to the controlled SCHEMA datasets are managed by data custodians of the SCHEMA consortium and the Broad Institute and are sent to sample contributing investigators for approval (see 12 for further details). Access to WTCCC2 control biological samples is managed and approved by the WTCCC (https://www.wtccc.org.uk/ccc2/). The Accession number for Sequence data from the Alzheimer's disease European sequencing project, which includes a subset of the Cardiff Alzheimer's disease cohort, is: dbGaP (phs000572.v7.p4 [https://www.ncbi.nlm.nih.gov/projects/gap/cgi-bin/study.cgi?study_id=phs000572.v7.p4] (stage 1)). SCHEMA case-control variants included in the current study are available for download through the SCHEMA browser (https://schema.broadinstitute.org/). Source data for Figure 1 are provided as a Source Data file.

Research involving human participants, their data, or biological material

Policy information about studies with human participants or human data. See also policy information about sex, gender (identity/presentation), and sexual orientation and race, ethnicity and racism.

Reporting on sex and gender	As part of our quality control procedure, we tested whether predicted sex from the genetic data matched genetic sex recorded in the new case-control data. We excluded samples if the genetic sex did not match the recorded sex. In our single gene tests, we also controlled for sex on X chromosome genes in our new case-control data. All details regarding sex, including the proportion of males and females included in our new cases and controls, are reported in the Supplementary Information.
Reporting on race, ethnicity, or other socially relevant groupings	Race, ethnicity, or other socially relevant groupings are not included in our study.
Population characteristics	We used principal component analysis to assess and control for population structure in the new case-control sample. We first grouped samples based on genetic similarity to the superpopulations used in the 1000 genomes (1KG) project, and excluded cases and controls that fell 4 standard deviations or more from the means of PCs 1 and 2 in samples predicted to be genetically similar to the 1KG European superpopulation reference. To ensure our findings are not confounded by population stratification, we performed a further PC analysis of these samples, and removed those that were outliers from on the largest PC cluster (see Supplementary Figure 2). A full description of our approach to control for population structure is presented in the Supplementary Methods.<br><br>In our gene-set analysis, we include the first 10 principal components, sex as defined by the genetic data, and the exome-wide burden of synonymous variation as covariates in our regression models. In our single-gene analysis, we stratified the SCHEMA cases and controls into 11 independent groups based on ancestry and exome capture platform as defined in the original SCHEMA study (Singh et al 2022). We also stratified the new cases and controls by genetically defined sex for genes on the X chromosome. A full description of how we adjust for population structure in our statistical analysis is provided in the methods and Supplementary Material.
Recruitment	Our study did not recruit new samples. The new case-control exome sequencing data was derived from previously recruited samples, all of which are described in the 'Sample description' section of the Supplementary Methods. Briefly, cases were derived from the previously ascertained CLOZUK2, CardiffCOGs, F-series and Affected-Sib cohorts. Controls were derived from WTCCC2 cohort, previously ascertained NCMH samples and samples from the Cardiff Alzheimer's disease cohort.
Ethics oversight	All research conducted as part of this study was consistent with UK regulatory and ethical guidelines. We gained National Health Service research ethics committee approval for the CLOZUK (10/WSE02/15) and Cardiff COGS (07/WSE03/110) studies. Multicentre and Local Research Ethics Committee approval was obtained for Cardiff F-Series, and all participants gave written informed consent to participate. For Cardiff Affected-Sib samples, written consent was obtained following local ethical approval guidelines. The control samples were recruited as part of independent projects, all of which have equivalent ethical permissions and data sharing procedures in place. Ethical approval for the NCMH sample obtained from Wales Research Ethics Committee 2 (reference: 16/WA/0323). REC numbers for the Cardiff AD project are 04/9/030 and 17/SS/0139.

Note that full information on the approval of the study protocol must also be provided in the manuscript.

Field-specific reporting

Please select the one below that is the best fit for your research. If you are not sure, read the appropriate sections before making your selection.

☒ Life sciences ☐ Behavioural & social sciences ☐ Ecological, evolutionary & environmental sciences

For a reference copy of the document with all sections, see nature.com/documents/nr-reporting-summary-flat.pdf

Life sciences study design

All studies must disclose on these points even when the disclosure is negative.

Sample size	Sample size was not predetermined in our study. We analysed the largest publicly available schizophrenia exome sequencing datasets from case-control and trio studies that were available at the time of study design. When combined with the new case-control sample, this resulted in the largest exome-sequencing study of schizophrenia at the time of submission.
Data exclusions	We only sequenced cases with a DSMIV or ICD10 criteria for schizophrenia or schizoaffective disorder, and controls without a known diagnosis of a psychotic disorder. In our new case-control dataset, we excluded samples based on low quality sequencing data, relatedness and population structure (full description in supplementary methods). We also excluded low quality variants and genotypes (full description in supplementary methods).
Replication	To maximise power for gene discovery, and therefore reproducibility, we meta-analysed all available case-control and trio data that were available at the time the study was conducted. We used our new case-control data to replicate genes previously implicated in schizophrenia at exome-wide significance or FDR < 5% (see Supplementary Table 8 and 10).
Randomization	Randomization was not applicable as this is an observational study.
Blinding	Blinding was not applicable as this is an observational study.

Reporting for specific materials, systems and methods

We require information from authors about some types of materials, experimental systems and methods used in many studies. Here, indicate whether each material, system or method listed is relevant to your study. If you are not sure if a list item applies to your research, read the appropriate section before selecting a response.

Materials & experimental systems

n/a	Involved in the study	
☒	☐	Antibodies
☒	☐	Eukaryotic cell lines
☒	☐	Palaeontology and archaeology
☒	☐	Animals and other organisms
☒	☐	Clinical data
☒	☐	Dual use research of concern
☒	☐	Plants

Methods

n/a	Involved in the study	
☒	☐	ChIP-seq
☒	☐	Flow cytometry
☒	☐	MRI-based neuroimaging

Plants

Seed stocks	*Report on the source of all seed stocks or other plant material used. If applicable, state the seed stock centre and catalogue number. If plant specimens were collected from the field, describe the collection location, date and sampling procedures.*
Novel plant genotypes	*Describe the methods by which all novel plant genotypes were produced. This includes those generated by transgenic approaches, gene editing, chemical/radiation-based mutagenesis and hybridization. For transgenic lines, describe the transformation method, the number of independent lines analyzed and the generation upon which experiments were performed. For gene-edited lines, describe the editor used, the endogenous sequence targeted for editing, the targeting guide RNA sequence (if applicable) and how the editor was applied.*
Authentication	*Describe any authentication procedures for each seed stock used or novel genotype generated. Describe any experiments used to assess the effect of a mutation and, where applicable, how potential secondary effects (e.g. second site T-DNA insertions, mosiacism, off-target gene editing) were examined.*
